# Substructural Approach for Assessing the Stability of Higher Fullerenes

**DOI:** 10.3390/ijms22073760

**Published:** 2021-04-04

**Authors:** Ayrat R. Khamatgalimov, Valeri I. Kovalenko

**Affiliations:** 1 FRC Kazan Scientific Center, Arbuzov Institute of Organic and Physical Chemistry, Russian Academy of Sciences, 420088 Kazan, Russia; koval@iopc.ru; 2 Department of Environmental Engineering, Kazan National Research Technological University, 420015 Kazan, Russia

**Keywords:** higher fullerenes, structure, stability, substructure, radical, overstrain

## Abstract

This review describes the most significant published results devoted to the study of the nature of the higher fullerenes stability, revealing of correlations between the structural features of higher fullerene molecules and the possibility of their producing. A formalization of the substructure approach to assessing the stability of higher fullerenes is proposed, which is based on a detailed analysis of the main structural features of fullerene molecules. The developed substructure approach, together with the stability of the substructures constituting the fullerene molecule, helps to understand deeper the features of the electronic structure of fullerenes.

## 1. Introduction

The molecular and electronic structures of fullerenes have been of great interest in modern science for over 35 years. On the one hand, this can be explained by the fact that a completely new form of carbon existence—molecular—has emerged; these closed carbon clusters, which have an intramolecular cavity, were able to enclose individual atoms, several atoms and even small molecules inside themselves and outside participate in the addition reactions. All this is new and unusual in terms of theoretical chemistry and fundamental science in general. On the other hand, the physical and chemical properties of these molecular systems suggest a wide potential for their practical use in creating new materials. The possible practical application of fullerenes as doping additives of various materials (including superconducting and ferromagnetic), in molecular electronic devices, catalysts, as medicines, current sources, molecular sieves, devices for gas storage and solar energy converters make the study of these objects extremely important.

Recently, about one thousand works a year have been published dedicated to the study of fullerenes. However, despite a number of remarkable discoveries in this field, there is a lack of in-depth and comprehensive research, both theoretical and practical.

Therefore, at the present, the most widespread and studied fullerenes are C_60_ and C_70_, which are quite accessible for research and practical purposes: there is a significant amount of structural and chemical information about them today. In contrast, studies of higher fullerenes are characterized by much fewer data available. Information on the nature of the distribution of bonds in the higher fullerenes and the influence of the spatial and electronic structure on their stability will undoubtedly be useful to predict their reactivity and properties.

The main problems in the study of the electronic structure of higher fullerenes that obeying to the Isolated Pentagon Rule (IPR) are related to their low availability, difficulties in separating and identifying isomers and, consequently, the small volume of experimental data obtained. However, the results of recent studies on the properties of carbon nanomaterials are quite optimistic.

The reasons of instability of higher fullerenes (both IPR and non-IPR) in the form of empty molecules and the stability of their endohedral and exohedral derivatives are not yet explained. A variety of criteria for evaluating the stability of different fullerenes have been proposed to address this issue; however, they do not always successfully explain the above situation, which makes it much more difficult to use them for discussion and/or prediction of the results of experiments.

Quantum chemical methods are used very successfully in studies of the structure of higher fullerenes, that makes them one of the most actively developing area. It is difficult to find the most closely related object and method of investigation than fullerenes and quantum chemistry; this combination is most often found in modern scientific literature, which shows that the application of quantum-chemical methods in the study of the electronic structure and stability of fullerenes is promising and actual task.

The majority of quantum chemical studies give an assessment of the stability of fullerenes based on two parameters: the minimal relative energy and the maximal energy of Highest Occupied Molecular Orbital (HOMO) and Lowest Unoccupied Molecular Orbital (LUMO) gap in a series of molecules of all isomers of this fullerene C_n_. In other words, we are talking about the integral characteristics of the molecule of this isomer, without detailing the geometric and electronic features of the structure of the molecule. In fact, much of the information obtained in the calculations remain out of sight of the researcher. This is partly due to the significant time (and patience) required to process and analyze a vast amount of data on bond lengths, valence and dihedral angles, electronic density distribution, etc.

In this review, we provide a deeper insight into the role of quantum-chemical calculations and particularly DFT methods, in identifying the nature of stability of higher fullerenes as well as the relationship between the structural features of the molecules of higher fullerenes with the possibility of their obtaining. Additionally, we review a formalization of the substructural approach to assessing the stability of higher fullerenes using DFT methods that provides an assumed structural formula with the distribution of single, double and delocalized bonds.

## 2. Stability Criteria for Fullerenes

The problem of predicting the relative stability and the possibility of the existence of fullerenes has over forty years of history starting with the works by E. Osawa et al. [1,2] and D.A. Bochvar and E.G. Galpern [3]. In 1987, H. Kroto formulated several simple empirical rules, the implementation of which is necessary to stabilize the carbon cages [4]:

1. In stable carbon clusters (fullerenes), each atom has a coordination number 3.
2. Clusters containing only five- and six-membered rings (pentagons and hexagons, respectively) are more stable.
3. Five-member cycles in carbon clusters must be isolated (the Isolated Pentagon Rule).
4. More symmetric carbon clusters are more stable, which leads to a uniform distribution of the curvature of the cluster surface.
5. It is necessary to have a closed electronic shell for a stable cluster.

Similar rules were proposed by Schmalz et al., in 1988 [5] during calculating more than 30 fullerenes. All subsequently developed stability criteria for fullerenes can be divided into two groups: criteria depending on the electronic structure and criteria related to geometric parameters of the molecule.

The first group of criteria for the prediction of stable fullerenes can include the aforementioned energy criteria of aromaticity which estimates the energy gain and, as a consequence, stabilization, as a result of cyclic delocalization. This is, for example, the resonance stabilization [6], which has been referred to “electronic effects”; stable structures were identified by the theory of Hückel molecular orbitals and the resonance theory [7]. When choosing the most stable structures, Taylor identified the structures with the largest number of hexagons containing alternating single and double bonds [8].

It should be mentioned that the topological studies based on Wiener indices and associated topological roundness were able to explain fullerenes stability. This original computational approach is based on the generalization of the topological efficiency potentials which may be used to study all types of carbon allotropes. In particular, the effects of topological efficiency in driving fullerene isomerization have been analyzed for the C_84_ IPR molecules [9,10]. The correlations between the stability/achievability of the C_84_ fullerene isomers and their topological and structural parameters (minimal extremal roundness, maximal sphericity and maximal volume) were revealed. An inverse correlation between the sphericity and extremal roundness has been found and explained. Both these quantities are shown to be efficient for predicting achievable fullerene structures [9,10]. The reported results revealed the two isomers C_84_ #24 (D_2d_) and C_84_ #1(D_2_) as the most stable molecules [9,10] that are synthesized in large amounts and hence, called major isomers (the numbering of the isomer is the one produced by PROGRAM FULLGEN [11]); these isomers are C_84_ #23 (D_2d_) and C_84_ #22(D_2_), according to [12]).

Diener and Alford suggested dividing fullerenes into two classes in terms of their stability [13]. Stable fullerenes have a large energy gap between the highest occupied and lowest unoccupied molecular orbitals (HOMO-LUMO); fullerenes C_60_ and C_70_ were attributed to them. The second class consists of unstable fullerenes that have either a small energy gap or have a radical structure with unpaired electrons. A similar division was also proposed based on the results of electrochemical studies of fullerenes [14]. Indeed, the large energy difference between the frontier orbitals means high stability of the molecule and its low reactivity since it is energetically unfavorable to add electrons to a high-lying HOMO and to donate electrons from a low-lying LUMO. However, the analysis of literature data showed that this parameter is not always sufficient to predict the stability of fullerenes [15,16]. Thus, as the size of the molecules increases, the ratio of hexagons to pentagons increases, leading to decreasing of the energy gap between the boundary orbitals (highly aromatic graphite has no forbidden zone at all). Therefore, in order to assess the kinetic stability of fullerenes, Aihara suggested correlating the value of the HOMO-LUMO energy gap with the number of carbon atoms in the fullerene C_n_ (criterion T) and supplementing it with a number of other parameters: for example, the resonance energy of the bond, which is a contribution of this π-bond into the topological resonance energy [17]. Assuming that the values of these parameters represent a measure of the stability of the molecule, Aihara supposed [17] that some fullerenes should be stable. However, it has not been explained why fullerene C_72_ or isomers 1 (D_2_) and 20 (T_d_) of fullerene C_84_ cannot be isolated, although they all satisfy the above criteria (Table 1).

The second group of criteria was proposed to include the “geometric” or “steric” factor, which takes into account the encirclement of a different number of hexagons in the fullerene (“neighbor index”) [6]. It has been suggested that the stability of all carbon clusters is affected by the number of nearest neighboring contacts, the curvature of the molecular surface and the number of adjacent pentagons [19]. Thus, in [20], the calculations of all possible 1812 isomers of fullerene C_60_, including non-IPR ones, were carried out and it was found that the three conjugated hexagons significantly increase the total energy of the molecule. The authors explain this by the appearance of local flatness, which in turn implies the formation of high strain in another part of the fullerene surface. The obvious thesis about the effect of local strains on the stability of fullerenes is consistent with many other authors; in particular, Haddon [21] argued that local strain is an important factor in the stability and reactivity of fullerenes, however, the authors failed to quantify it (except for the value of relative total energy).

According to Raghavachari et al. [6], there is a competition between the electronic and steric factors: for example, the presence of coronene substructures leads to high thermodynamic stability due to the effect of resonance stabilization; however, steric factors raise the overall energy of the system.

Novel topological approach dealing with higher fullerenes and their chiral isomers is proposed by K. Balasubramanian [22,23]. The combinatorial generation function methods that combine Mȍbius inversion and character cycle indices for the enumeration of stereo, position and chiral isomers of icosahedral giant fullerenes are developed and used for polysubstituted C_180_ and C_240_ giant fullerene cages. The prediction of a number of sites with different symmetry in the fullerene cage can also aid in the future computations of potential minima of small molecules trapped inside giant fullerene cages. A similar combinatorial technique was applied to face colorings of higher and giant icosahedral fullerenes C_80_, C_140_, C_180_ and C_240_. This enumeration of face colorings, especially with restrictions to either hexagons or pentagons can aid in future studies on isomerization reaction graphs arising from the movements of pentagons or hexagons through various mechanisms including a series of Stone–Wales transformations that can produce other giant fullerene isomers.

Thus, the criteria previously proposed for describing the stability of fullerenes are often unable to explain completely the instability of some fullerenes. Moreover, in several cases, there is a discrepancy between the proposed criteria and the experimental data. Apparently, a detailed analysis of the structure of fullerene molecules is necessary from the point of view of the stability of substructures, the combination of them makes up a fullerene molecule. The synthesis of fullerenes is believed to follow a “bottom-up” mechanism [24]. At the same time, the synthesis of fullerenes is also possible using the “top-down” mechanism, when the formation of a molecule occurs as a result of folding a graphene sheet in a beam of electrons [25] or in space [26]. Apparently, the time has not yet come to judge the subtleties of obtaining fullerenes. It is important to emphasize that a number of isomers that cannot be obtained as empty structures; however, f.e. pristine fullerenes may be easily obtained as endohedral fullerenes. Let us recall that the process of obtaining fullerene from graphite takes place in electric arc plasma, where electrons, metal cations, etc., are present [27]. One can imagine that this can create a fragment of a fullerene molecule in the form of an anion, which attracts a metal cation and then the fullerene cage closes. In other words, an ionic pair, a precursor of endohedral fullerene, is formed. This assumption is supported by the fact that in the overwhelming majority of cases the process of obtaining fullerenes in the electric arc takes place in a helium atmosphere, but the probability of obtaining the endohedral fullerene He@C_n_ is very low [28]. Obviously, there is no driving force that would hold the helium atom near the growing molecule, in contrast, to the formation of the ionic pair. It is well known that helium atoms can be introduced into the already obtained fullerene molecules under very harsh conditions (600–650 °C, 2−3 × 10^3^ atm) [29,30,31,32].

Thereby, the existing problems in the study of the relationship between the structure and stability of higher fullerenes require an approach that will connect the structural features of higher fullerene molecules with the possibility of their obtaining, the main principles of which will be presented in the next chapter.

## 3. Basic Principles of the Substructural Approach to Modeling the Structure and Stability of Higher Fullerenes

**Figure 1:**
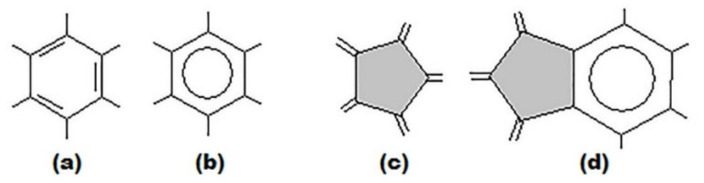
The main structural elements of the fullerene molecule: hexagon with single and double bonds alternation (**a**), hexagon with π-bonds delocalization (**b**), pentagon (**c**), pentagon adjacent to hexagon with delocalized π-bonds (**d**).

To solve the above problems of assessing the stability of higher fullerenes, a approach to modeling the structure and stability of higher fullerenes that connects the structural features of higher fullerene molecules with the possibility of their obtaining was developed [33,34,35,36,37,38]. The development of the approach began with a thorough analysis of structural parameters of the molecules of two fullerenes C_60_ and C_70_, which are the most stable and best studied among all known fullerenes. Based on the results of structural experimental methods [39,40], the arrangement of bonds was clarified. Thus, for C_60_ it was found that pentagons consist only of single bonds and in hexagons there is an alternation of double and single bonds (Figure 1a,c). At the same time, it should be noted that the correct arrangement of bonds in C_60_ molecule was theoretically determined in 1973 [3]—12 years (!) before the discovery of buckminsterfullerene. As for the structure of C_70_ fullerene molecule, it turned out that the bond distribution at the poles of the molecule is the same as in the case of C_60_ fullerene. However, the five hexagons at the equatorial belt of the molecule have nearly equal bond lengths, i.e., they are closer to benzene: their bonds are π-delocalized [38,40,41].

The semiempirical substructural approach to modeling the structure of IPR fullerene molecule consists of three stages:

**Stage 1.** Creation of structural formula of a fullerene molecule.

It is known that the topology of any C_n_ fullerene molecule determines only the mutual position of 12 pentagons and (n/2–10) hexagons [12] without specifying what type of bond exists between any pair of C-C carbon atoms in a given molecule. The structural formula of any fullerene molecule as generally accepted in organic chemistry consists of the arrangement of single, double and delocalized π-bonds. Within the framework of the chemical graph theory, it means replacing a simple graph with a chemical pseudograph [42].

In this case, the main structural elements are: (i) hexagon with alternating single and double bonds (Figure 1a), (ii) hexagon with delocalization of π-bonds (Figure 1b), (iii) pentagon with single bonds (Figure 1c) and (iv) pentagon adjacent to a hexagon with delocalized π-bonds (Figure 1d).

It is necessary to take into account a very important requirement: the symmetry of the fullerene molecule should be retained after the arrangement of the bonds. The Atlas of Fullerenes [12] presents the calculated parameters of ^13^C NMR spectra for fullerenes from C_20_ to C_50_ and IPR isomers from C_60_ to C_100_: the number of signals and their relative intensities strictly determined by the symmetry of a given molecule; it means that all carbon atoms in C_n_ fullerene molecule form some groups of equivalent atoms in it. The analysis of published data showed that there was not any experimentally obtained ^13^C NMR spectrum of IPR pristine fullerenes that did not satisfy this rule. As a consequence, it became possible to identify a number of fullerene isomers only based on ^13^C NMR spectral measurements. Thus, preservation of the symmetry of a molecule must be strictly obeyed in the arrangement of the different types of bonds.

**Stage 2.** Identification of substructures, i.e., fragments, of a fullerene molecule that are characterized by their geometry and electronic parameters.

The analysis of the structure of the fullerene molecule as a set of its constituent substructures proved to be very fruitful. We have shown that the rule of characteristicity of substructures is realized: the substructure mainly retains its geometric and electronic characteristics regardless of which fullerene it belongs to. It was shown that fullerenes have only a slight global delocalization of π-bonds [43] due to the curvature of the fullerene cage, which leads to the preservation of properties of single and double bonds; therefore, fullerenes in reactions behave like olefins [44]. Indeed, the substructure is connected to the remaining part of the fullerene molecule (or separated from it) by single bonds, which are poor conductors of electron density.

**Figure 2:**
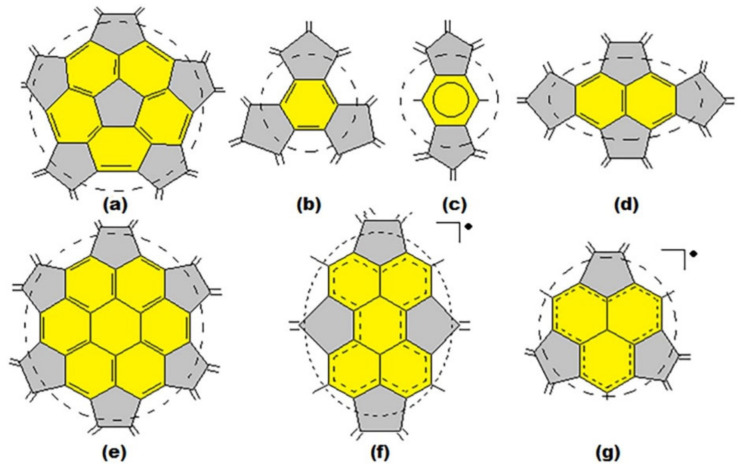
The main substructures of the fullerene molecule: (**a**) corannulene, its component is sumanene (**b**), s-indacene (**c**), naphthalene (**d**), coronene (**e**), perylene (**f**) and phenalenyl-radical (**g**). Dotted circles are crossed single bonds around the substructure.

The most characteristic substructures and frequently encountered in molecules of the IPR fullerenes starting with most stable fullerenes are the corannulene substructures of the whole C_60_ and at the poles of C_70_ as well as s-indacene at the C_70_ equatorial belt (Figure 2a,b); the sumanene and naphthalene substructures are the parts of corannulene substructure (Figure 2c,d); then, coronene (Figure 2e), perylene (Figure 2f) and phenalenyl substructures (Figure 2g); the names of substructures correspond to their non-fullerene predecessors in organic chemistry. It is easy to see that they are all separated off (or connected with) the rest of the molecule by single bonds. In the arrangement of bonds, these substructures (corannulene and s-indacene) were first of all considered as the most preferable in the analysis of a structure of molecules of the other higher fullerenes because they belong to the most stable fullerenes.

**Figure 3:**
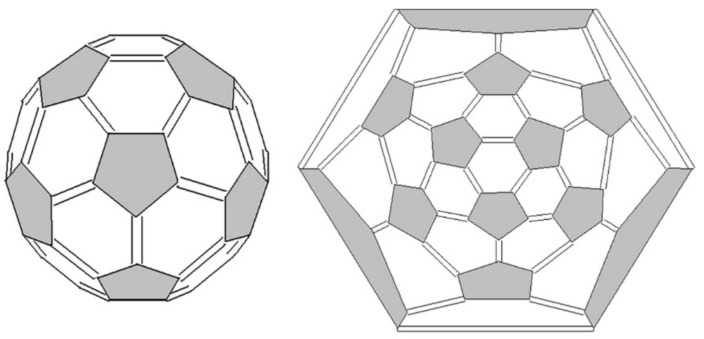
3D-model and Schlegel diagram of C_60_ (I_h_) fullerene.

The known detailed molecular structures of the most available and stable IPR fullerenes C_60_ and C_70_ that served as the basis for the proposed approach, fully satisfy the above considerations: all pentagons consist of single bonds and in hexagons there is an alteration of single and double bonds. Double bonds in C_60_ are mutual for a pair of hexagons, while single bonds are mutual in a hexagon-pentagon joint. At the same time, as can be seen, the position of the bonds does not reduce the I_h_ symmetry of the molecule (Figure 3). The unique stability of fullerene C_60_ is determined by its highest symmetry and, as a consequence, by the uniform distribution of a significant strain (0.41 eV/atom) over the molecule in comparison, for example, with the isomer 1 (D_2_) of fullerene C_84_ (0.40 eV/atom [45]), the last one is unstable and has not been obtained [36], vide infra.

**Figure 4:**
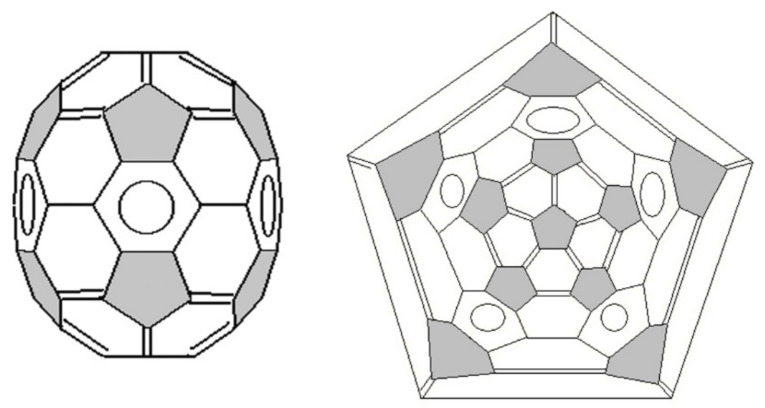
3D-model and Schlegel diagram of C_70_ (D_5h_) fullerene.

**Figure 5:**
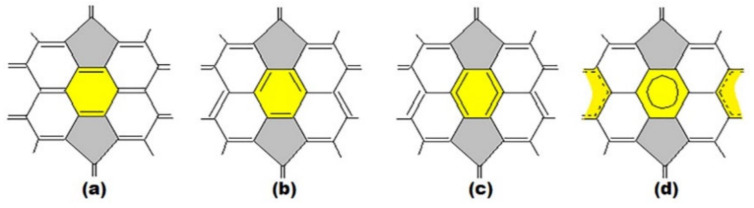
(**a**–**d**)—represents various options of bonds distribution in indacene substructure.

In the molecule of fullerene C_70_, the appearance of ten additional atoms, compared to fullerene C_60_, leads to a decrease in symmetry to D_5h_ (Figure 4), nevertheless, high strain in the molecule (0.38 eV/atom [45]) is distributed fairly evenly. The position at the poles of two corannulene substructures is similar to fullerene C_60_. However, the requirement to follow the above rules for bonds arrangement leads to the appearance of five hexagons with delocalized π-bonds on the equatorial belt, i.e., the s-indacene substructures (Figure 5d), because the first two options violate the requirement that pentagons consist only of single bonds (Figure 5a), as well as the requirement to preserve the symmetry of the molecule (Figure 5b). The third option (Figure 5c) violates the tetravalence of the carbon atom. The only option that satisfies the above conditions (Figure 5d) ultimately forms the structure of fullerene C_70_ that contains five hexagons with delocalized π-bonds on the equatorial belt (Figure 4) [37,38], which is in good agreement with the experimental data [40]. Such a variation in the distribution of bonds eliminates the questions about the possibility of pentavalent carbon in the equatorial belt of C_70_, which was previously considered in [8] and it also indicates an inaccuracy in the distribution of bonds in C_70_ [44]. Note also that the X-ray data confirm the presence of delocalization in the central hexagon in s-indacene derivatives and the s-indacene structure has a flat planar form [46,47].

Thus, based on the rule of preference of corannulene (Figure 2a) and s-indacene (Figure 2c) substructures, which are characteristic for the most stable fullerenes C_60_ and C_70_, the distribution of different types of bonds in the unknown fullerene should start strictly with the observing the symmetry of the molecule. Let us now examine the coronene substructure (Figure 2e), which represents seven symmetrically arranged hexagons. This substructure is flat, while the fullerene shell tends to be spherical. Therefore, the coronene substructure, as well as similar structures representing flat fragments consisting only of hexagons, is also likely to be destabilizing due to excessive local strains in the molecule.

**Figure 6:**
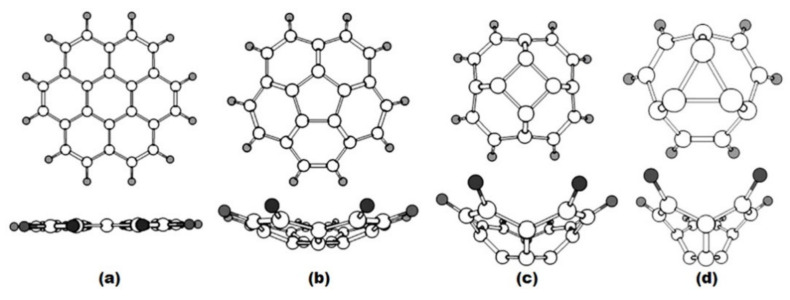
Optimized structures of molecules with six- (**a**), five- (**b**), four- (**c**) and three-membered (**d**) cycles surrounded by hexagons [48].

It should be emphasized that the corannulene substructure has a curvature that fits into sphere-like molecules of fullerenes C_n_ (n ≥ 60). This is well illustrated by the quantum-chemical optimization (B3LYP/6-31G*) of geometric parameters (bond lengths, valence angles, sums of valence angles, dihedral angles) of a number of model structures in which the molecular strain consistently increases (structures with six—(coronene), five—(corannulene), four—and three-membered central cycle surrounded by hexagons (Figure 6)). It was shown that coronene itself (Figure 6a) has the flat structure of seven symmetrically condensed hexagons, while the structures of c–d (Figure 6) are non-planar [48]. The discovered alternation of bonds in hexagons of corannulene and coronene substructures is consistent with earlier theoretical (B3LYP/6-31(d)) and experimental (including X-ray structural analysis) studies [49,50,51,52].

When moving from coronene to corannulene structures and further to the structures with four- and three-membered cycle, computations predict a decrease of the sum of valence angles at the carbon atom located at the top of the cycle and dihedral angles between the planes of two hexagons **I** and between the planes of the hexagon and the corresponding central cycle **II** (see Table 2). This clearly shows that the local strains are the least pronounced in corannulene and coronene. Dihedral angles in the surrounding hexagons are not shown because, as it can be seen from Figure 6, the deformation or curvature is noted in corannulene and the structures with four- and three-membered central cycles. Such a distortion was marked [50] by the main difference of corannulene molecule C_20_H_10_ from the corannulene structure in C_60_ fullerene where the hexagons and pentagons are planar. Similar studies of a series of polyhedrons with four-, five-, six- and seven-membered cycles [53] led to the creation of a model for assessing the stability of a molecule based on the relationship between the binding energy and geometric parameters. It was shown [54] that the stability of a carbon cage with a triangular cluster is impossible because of the large σ-strain.

**Figure 7:**
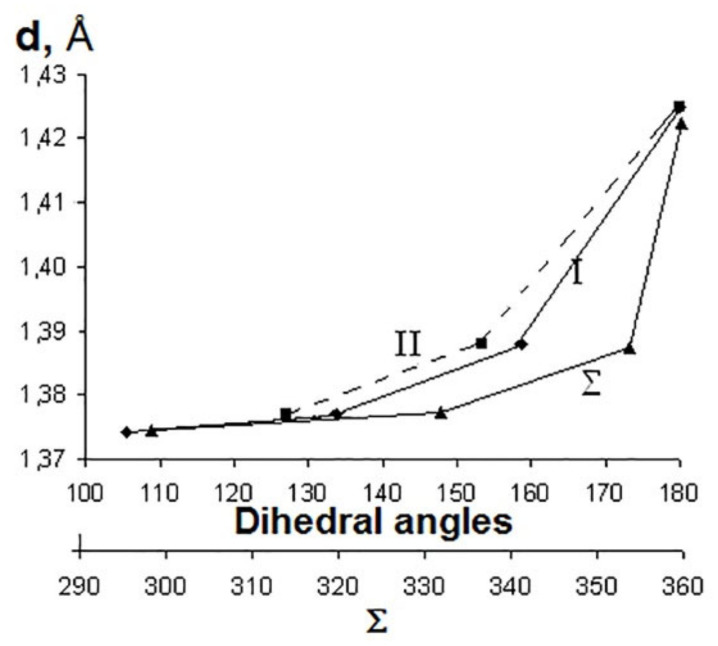
Correlation between double bond length **d** and values of the dihedral angles (I, II) and the sums of valence angles (**Σ**) for structures with six-, five-, four- and three-membered rings [48].

Analysis of the relationship between the length of the double bond (CC_radial_) and the corresponding values of dihedral angles, as well the sum of valence angles in the presented structures, allowed us to observe another clear correlation (Figure 7); a decrease in the values of dihedral angles and the sum of valence angles (and, therefore, an increase in local strains) is accompanied by shortening of the radial double bond.

**Figure 8:**
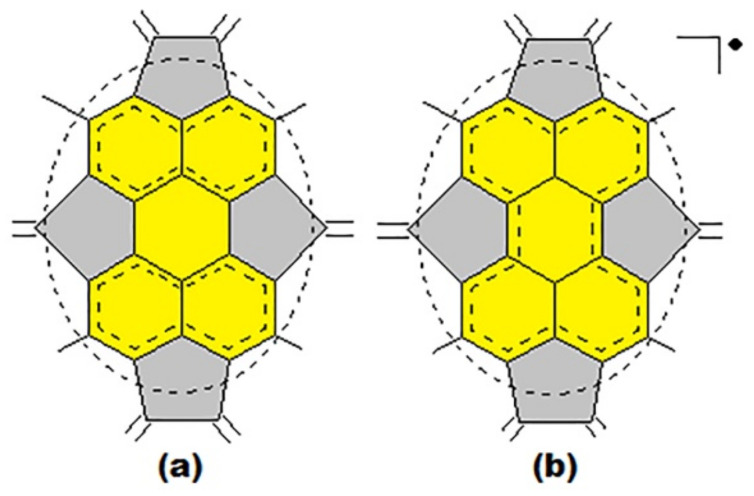
Perylene substructure with closed (**a**) and open (**b**) electron shells. Dotted ovals indicate single bonds that separate (bind) perylene to the rest of the molecule.

In the perylene substructure, according to its C_2v_ symmetry, two types of bond distribution, with a significantly different character of the fullerene electron shell, are possible (Figure 8): the first type (Figure 8a) is characterized by a closed electron shell; on the contrary, the second one (Figure 8b) has an open electron shell. Apparently, the nature of the electron distribution of this substructure will depend on the environment of the substructure in each specific fullerene isomer and can be determined by quantum chemical calculations.

**Stage 3.** At last, carrying out quantum-chemical calculations of a given molecule.

On the one hand, such calculations allow assessing the correctness of the prior arrangement of bonds and the construction of the structural formula of the molecule. On the other hand, such calculations could lead to the same results without the first two stages, but with huge expenditures of time and intellectual efforts to process huge amounts of computational information.

An important result of a priori analysis of the structural features of the fullerene molecule is the identification of radical substructures, i.e., the identification of substructures that may have unpaired electrons. This means that quantum-chemical calculation of such a molecule should be carried out with taking this circumstance into account, keeping in mind that open-shell is a direct indication of the probable instability of this fullerene. When the arrangement of bonds leads to complete satisfaction of all requirements of this approach, it means that all valence electrons in the molecule are paired (closed-shell). For example, all carbon atoms in C_60_ (I_h_) and C_70_ (D_5h_) molecules meet all the requirements of the approach, but in the structure of molecules of fullerenes C_74_ (D_3h_) and C_76_ (T_d_) inconsistencies are immediately detected. Thus, the phenalenyl substructure (Figure 2g) is present in both of these molecules: two in the first and four in the second, respectively. All of them are located symmetrically on the axes of symmetry of the third order in both molecules. Consequently, they formally have a valence of 3; any other option implies violation of the requirement to preserve the symmetry of the molecule. This suggests that we are dealing with a phenyl radical (which is in good agreement with the non-fullerene analogs of this substructure [55,56,57,58,59]). Our calculations of these fullerenes confirmed the expectation of the radical nature of their molecules [59,60,61,62,63,64,65], as well as their behavioral features according to experimental data [13,66,67,68].

**Figure 9:**
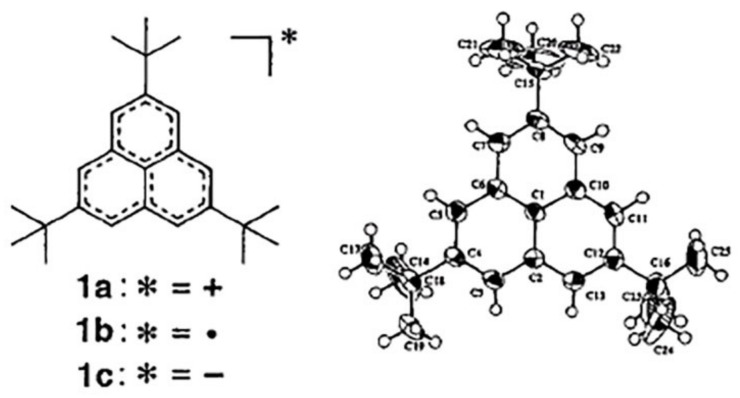
Molecular structure of 2,5,8-tri-*tert*-butyl-phenalenyl radical. Reprinted with permission from [56]. Copyright © 2021, American Chemical Society.

The analogs of fullerene substructures are widespread in organic chemistry. The phenalenyl substructure is no exception and has a well-known analog: the phenalenyl radical, which is an unstable compound. However, its derivative, namely, 2,5,8-tri-*tert*-butyl-phenalenyl radical (Figure 9) is already a stable crystalline compound [56].

**Figure 10:**
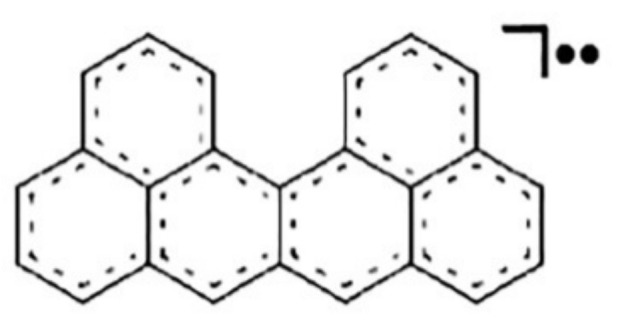
Uthrene structure. Reprinted with permission from [58]. Copyright © 2021, Royal Society of Chemistry.

It is important to note here that the crystallization of this derivative takes place in the form of a centro-symmetric π-dimer with the angle between two molecules 60° to reduce the steric interactions of tret-butyl groups. Such stabilization of dimers is well explained by their proposed radical structure, which was also noted in the calculations of compounds containing two phenalenyl radical fragments, which showed a small size of the HOMO-LUMO energy gap leading to a singlet biradical character under normal conditions and activated triplet biradical [57]. The singlet biradical character was also confirmed by X-ray crystallographic analysis showing the existence of dimer pairs with short contacts (3.1 Å). A similar biradical structure is also characteristic for the uthrene molecule C_24_H_14_ (Figure 10), which, according to calculations [58], has a triplet configuration. Such substructures made up of several simple substructures, for example, from four “fused” phenalenyl radicals, are also observed in fullerenes [59].

According to the calculations of HOMO-LUMO gap of phenalenyl-like structures (Table 3) the systems are stabilized upon accepting one electron and, conversely, attaching a second electron, which is obviously redundant, destabilizes these systems [34]; the results of the study are in complete agreement with the experimental X-ray structural analysis [56]. The reason for this stabilization can be revealed by analysis of the correlation between the lengths and orders of π-bonds in polycyclic aromatic systems and fullerenes [69] (see Figure 7). It is obvious that the substitution of hydrogen atoms in the phenalenyl radical lowers the electron density at the periphery of the molecule and leads to some stabilization of the radical. Continuing this analogy, one can expect that this kind of stabilization will take place in fullerenes as well.

A preliminary analysis (before quantum-chemical calculations) and formation of the structural formula of fullerene molecule within the framework of the proposed approach is very rational. In addition, our experience of such formations has shown the effectiveness of the “fullerene molecular constructor”. It should be noted that, so far, there has never been a failure in such an analysis, although there are undoubtedly limits to the applicability of this approach, which is the subject of our further research.

Thus, we have described the main substructures found in higher fullerenes. However, the possible set of substructures is not limited to the basic one presented in this chapter. With an increase of the number of carbon atoms and, accordingly, the number of hexagons in the structure of fullerene molecules, other substructures will inevitably appear, but all of them can be characterized from the point of view of the nature of the electron shell as well as the presence of possible excess local strains and, accordingly, the effect on the stability of some isomer of fullerene. Their characteristic feature is that they are all connected with the rest of the molecule by single bonds along which the transfer of electron density is limited. Therefore, substructures retain the features of their electronic structure regardless of the fullerene they belong to. An illustrative example of their isolation is the schemes of currents proposed in [70,71], which demonstrate well the differences between hexagons and pentagons together with their surroundings, expressed, as a consequence, in their different local aromatization.

Indeed, a similar analysis of the bond distribution in higher fullerenes C_72_–C_90_, C_104_ [33,34,35,36,59,60,61,62,63,64,65,69,72,73,74,75,76,77,78,79,80,81,82,83,84,85,86], including non-IPR [87,88,89,90,91] and small fullerenes C_n_ (n < 60) [92,93,94], confirmed that the instability of fullerenes may depend on two most important factors: (i) the presence of unpaired electrons in a molecule (i.e., an open electron shell), (ii) the excessive local strain of the molecule due to its topology.

The identified structural formulas of fullerene molecules with the complete distribution of all types of bonds (single, double and delocalized) are, in fact, the new information that allows the development of ways of chemical modification of fullerene molecules to obtain various derivatives. The substructural approach also makes it possible to predict ways to stabilize the higher fullerenes. For example, the reason of instability of fullerene C_74_ is its biradical structure [33,34,60]; therefore, its stabilization can be achieved by transformation into a structure with a closed electron shell (hydrogenation, halogenation, formation of a polymeric form, etc.) [62,63,64,65,75].

## 4. Methodological Features of Quantum Chemical Research of the Electronic Structure and Stability of Higher Fullerenes

The wide possibilities of quantum chemical calculations in solving specific problems of studying the structure of fullerenes have already been mentioned in the first chapter. The examples given have clearly demonstrated the importance of these studies since in some cases they allow to resolve controversial issues and draw conclusions that could not be drawn based on experimental studies.

Quantum-chemical calculations provide information on the structure, physical and chemical properties, the reactivity of organic compounds, including intermediates and transition states. No significant study of the structure of molecules or determination of the relationship between properties of molecules and their structure is possible without quantum-chemical calculations [95]; however, they are limited by computational power and accuracy of the methods used. Nevertheless, the development of a new generation of high-performance computational technologies and related software has made it possible (and already almost routine) to calculate very complex molecules. The accuracy of such calculations is quite comparable with the accuracy achieved in experiments [95,96].

Without pursuing the goal of describing the modern methods of quantum chemistry, we note that detailed information on modern achievements of quantum chemistry and its methods can be found in numerous reviews and monographs [see, for example, [95,96,97,98,99,100,101,102,103].

Some of the first simplified calculations of carbon clusters were made back in the 1960s, in particular by Pitzer and Clementi and later by Hoffmann, which have been reviewed by Z. Slanina [104]. Quantum-chemical models of carbon clusters described in the literature published before 1985 were based on classical polyhedrons–dodecahedron and truncated icosahedron. It should be mentioned that the possibility of the existence of a highly symmetric carbon molecule C_60_ [3] was also for the first time predicted by quantum chemical calculations. The π-electron model was applied to C_20_ and C_60_ carbon clusters [3], which proved itself well in describing planar conjugated hydrocarbons and their heteroanalogues [105].

There is a generally accepted scheme for quantum-chemical calculations of fullerenes [104]. First, all topologically possible structures are generated, which are, in fact, different variations of mutual arrangements of pentagons and hexagons on the fullerene surface. Although different topological algorithms are giving the same results [106], the most frequently used data are those published in the Atlas of Fullerenes [12], which presents the most popular spiral algorithm for generating fullerene topology.

Among all possible isomers, fullerene IPR isomers are the most frequently used for further studies. As already mentioned, structures in which each pentagon is surrounded only by hexagons will have the lowest relative total energy values, although exceptions to this empirical rule are possible in the case of fullerene derivatives. However, even if we only consider the IPR structures, in most cases several isomers will have relatively low energy. Then, in order to reduce the computational costs during the initial evaluation, as a rule the geometry optimization is carried out first using the methods of molecular mechanics or semi-empirical methods [107]. It was shown that both methods of molecular mechanics (MM3) [107] and semiempirical methods (MNDO, AM1, PM3) [108,109] are quite adequate for calculating the relative energies and nucleus-independent chemical shifts (NICS) of fullerenes. However, further structures should be re-optimized at a higher level.

In the recent years, the methods of density functional theory (DFT), which provide a quantum-mechanical description of atomic and molecular systems in terms of electron density, have become widely used, since the results in many cases are close to experimentally obtained values [97]. A peculiar empirical approach consists in the use of so-called hybrid functionals, in which a fraction of precisely calculated energy and the parameters for better reproduction of thermochemical properties of a set of molecules is used.

The DFT approach is widely used in the fullerene studies. In this context the methodological work by Rostami et al. [110] should be mentioned. Some electronic properties (LUMO, IP, HOMO, HOMO-LUMO gap and optical energy gap) of different C_60_ and C_70_ derivatives were evaluated using 20 density functionals and were compared with the available experimental data. It is shown that the functionals with a large %HF exchange significantly overestimate the HOMO-LUMO gap and are not suggested for electronic calculations. All non-hybrid functionals underestimate the value of HOMO in comparison to the experimental ionization potential (IP_e_), whereas in the hybrid functionals, the HOMO level becomes more stable by increasing %HF exchange and even in some functionals with a large %HF, HOMO overestimates the IP_e_. It is found that density functionals with somewhat a small %HF (~20–30%) such as B3LYP are more appropriate for electronic calculations compared to the other density functionals.

The most commonly used density functional method is the hybrid B3LYP (Becke’s 3-parameter hybrid method using the correlation functional of Lee, Yang and Parr) [111,112], which defines the exchange functional as a linear combination of various exchange contributions: Hartree–Fock’s, local and gradient-adjusted [100]. The choice of the DFT method, in particular B3LYP, is caused by the fact that it is one of the most frequently used methods for calculating the electronic structure of fullerenes, which allows easy comparison of the results of different research groups.

Chen and Thiel reported the assessment of the reliability of semiempirical methods MNDO, AM1 and PM3 and the density functional B3LYP/6-31G* on the base of a comparison of the results for the structures of 153 isomers of fullerenes and it was shown that the relative total energies are reproduced quite well [108]. The statistical evaluation of these data demonstrates that the quadratic correlation coefficient for higher fullerenes lies within the range of 0.86–0.92 and standard deviations are 5.16–6.46 kcal/mol.

Calculations of the relative total energies of fullerene isomers performed at the semiempirical level and by the DFT method are qualitatively consistent, the structural parameters predicted by the semiempirical method are quantitatively reproduced the results obtained by a more demanding DFT with the use of B3LYP functional [109]. Thus, a comparison of the bond lengths in fullerene C_70_ calculated by different methods and determined experimentally showed their good agreement. This means that all theoretical methods provide sufficiently justified parameters for higher fullerenes, in particular, for fullerene C_70_. At the same time, it is indicated that DFT calculations with the B3LYP functional have become a “de facto standard” in the calculation of fullerene isomers [109].

DFT methods have been shown to be the effective choice for calculating structural parameters of higher fullerenes [113,114]. Thus, the calculation of 16 fullerenes by different methods showed little difference in the bond lengths predicted by HF, DFT (BP and B3LYP functionals) and MP2 methods. The authors note that the non-empirical HF method overestimates the lengths of double bonds, while MP2 and DFT methods give comparable results. Therefore, in their opinion, DFT calculations are the best choice for determining the structural parameters of fullerenes because they require lower computational costs compared to the perturbation theory calculations [113,114,115]. Moreover, as was shown for a number of trifluoromethyl derivatives of various fullerenes [116,117] DFT optimized structures precisely correspond to the X-ray structural analysis data for C-C bond lengths of the carbon cage itself as well as for F_3_C…CF_3_ and F…F distances of the neighboring CF_3_ groups located in a para-position of one hexagon. In addition, to the bond lengths and distances between close contacts, the F-C-C-C torsion (dihedral) angles, which determine the conformations of CF_3_-groups in relation to the fullerene molecule, are correctly reproduced.

Special attention should be paid to the type of electron shell of the objects under study: open or closed, which affects the nature of the results obtained.

Thus, some general conclusions can be drawn. A rather unusual situation has arisen in the structural chemistry of fullerenes. At the present, an extensive material of experimental and theoretical studies of the electronic structure of fullerenes and their endohedral analogs has been accumulated. However, the overwhelming majority of studies have been performed for fullerenes C_60_ and C_70_: there is already a significant amount of experimental data on geometric parameters such as bond lengths, valence angles, etc.; their electronic and structural characteristics have been calculated using quantum chemistry methods.

For the higher fullerenes, the amount of the available data is much smaller. Except for fullerenes C_60_ and C_70,_ there was no traditionally accepted system for identifying bonds in fullerene molecules. In other words, currently, there is no generalization of structural information that is very useful for the chemistry of fullerenes such as the positions of single, double and delocalized π-bonds in fullerene molecules. Obviously, this lacuna makes it much more difficult to study the reactivity of fullerenes and their stability. In fact, the chemistry of higher fullerenes is grappling—nobody knows, which positions (bonds) of the fullerene cage would be involved in a particular reaction and why. To solve these problems the above new approach to modeling the structure and stability of higher fullerenes was developed.

At the same time, both experimental [118] and theoretical [119] attempts to predict the synthesis of different fullerenes (pristine or derivative) are continued. Such efforts may be unsuccessful because there is no knowledge clearly indicating which isomer of a fullerene molecule cannot be objectively obtained. Although the fruitfulness of the application of quantum-chemical methods for addressing a wide range of issues related to the electronic structure of fullerenes is beyond doubt, significant difficulties arise in determining the relationship between the electronic structure and stability of fullerenes. A large number of theoretical calculations of the electronic structures of fullerenes and endohedral metallofullerenes have been performed, but many problems require additional analysis.

## 5. Applying the Substructural Approach to Modeling the Structure and Stability of Higher Fullerenes

The stability or resilience of higher fullerenes is still a serious problem in modern physical chemistry. A comparison of the published results of experimental works and theoretical calculations clearly shows the existence of a correlation between them: experimentally obtained fullerenes are the most energetically advantageous among all possible isomers of a given series of fullerenes. The mutual arrangement of pentagons that set the curvature of the surface of the fullerene molecule, the presence of condensed hexagons, the electronic effects—all this together determines the total energy of the fullerene molecule and its stability or instability. This is very encouraging as it is a good test of stability. Therefore, quantum chemical calculations are used first to understand which fullerenes are stable and which are not. Despite the differences in accuracy and predictive power of semiempirical and non-empirical calculations (with and without correlation, on the minimum or extended basis), the order of relative stability of the isomers of one fullerene is the same in most cases. However, a comparison of relative values of total energies for any fullerene *C_n_*, which has, say, *m* IPR isomers allows only to estimate the probability isolation of isomers. At the same time, for fullerenes that have only one IPR isomer, one simply has to state that a given fullerene (for example, C_60_ or C_70_) has such total energy.

Moreover, it is was shown that the entropic part of the Gibbs energy becomes important at high temperatures [120,121] and calculations predict for some cases the most populated structure at higher temperatures to be not the one with the lowest potential energy. So, relative populations of higher fullerenes isomers of Eu@C_84_ and Eu@C_86_ were computed using the Gibbs energy based on potential energy from density functional theory calculations in order to evaluate their relative populations at higher temperatures, consistently using both enthalpy and entropy components of the Gibbs energy. The calculations in the floating encapsulate model treatment at temperatures around 1500K confirm that the recently isolated Eu@C_2_(13)-C_84_ and Eu@C_1_(7)-C_86_ species are major isomers in a relevant temperature region [120,121]. Similar dependence of relative concentrations of fullerene isomers on temperature were shown by quantum-chemical calculations for pristine fullerenes too, including small fullerene C_50_ (see, for example, [122,123,124,125,126]). These results represent another evidence of the importance of the Gibbs-energy treatment for evaluations of higher fullerenes stabilities.

For example, eleven isomers of C_84_ have already been isolated and identified. According to the calculations [78,79,127,128], all of them are located in the range between 0 and 25 kcal/mol (relative to the most favorable by energy IPR isomer 23 (D_2d_)). Assuming that this interval, which includes the values of relative total energies of all synthesized isomers and is equal to 25 kcal/mol, can be taken as some criterion of stability and transferred to other higher fullerenes, it turns out that the number of energetically stable fullerenes from C_70_ to C_84_ should be much greater because the difference between the relative total energies of stable and unstable isomers of other fullerenes is much smaller. However, they do not exist and it is not known whether they can be obtained at all.

**Figure 11:**
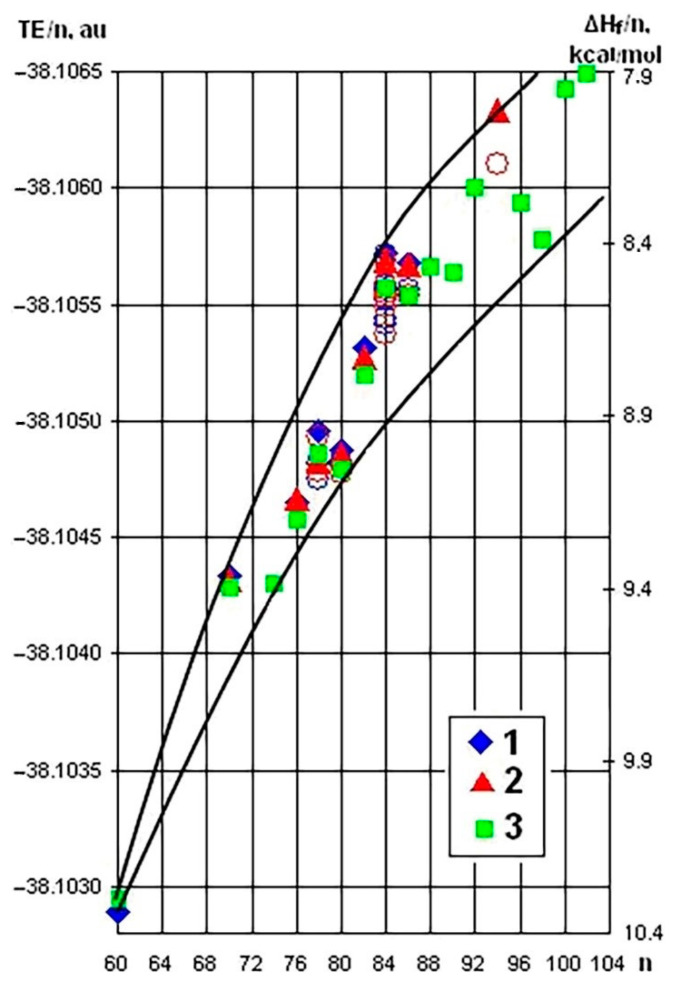
Total energies (TE/n) and standard enthalpies of formation (ΔH_f_/n) per one carbon for the most stable IPR isomers of C_n_ fullerenes (**1**—B3LYP/6-31G* [129], **2**—B3LYP/6-31G [35,69], **3**—B3LYP/6-31G* [108,130]). The remaining produced, extracted and characterized isomers are shown by a circle.

This question—why for some fullerenes several isomers were obtained and for others their number is less, while the difference in energy between the obtained isomers in the first case exceeds this value for the others—has not yet received an answer. Nevertheless, the array of available data allows us to make some comparisons and an attempt to move forward along this path. First, let’s look at the generalized picture that connects the stability of fullerenes and their total energies. For this purpose, we will use the given total energies and standard enthalpies of formation per one carbon atom (TE/n and ΔH_f_/n) [35,69] (Figure 11).

As a result, a certain selected sector or “beam of stability” was formed. Its upper border is determined by the minimum energies TE/n (and a heat of formation ΔH_f_/n). The most stable fullerenes C_60_, C_70_, C_84_ (isomers 23 (D_2d_) and 22 (D_2_)) and isomer 133 (C_2_) of fullerene C_94_ are located there. The lower curve defines the border of maximum values of energies and heats of formation for all known stable isomers of fullerenes C_n_, with the instability region located below it.

First of all, attention is drawn to the steady tendency towards a decrease in the rate of energy growth with an increase in the number of atoms and, as a consequence, the size of the fullerene shell. Obviously, it reflects the general decrease in a strain of a sphere-like molecule moving from C_60_ to higher fullerenes. It should be noted that the molecules of C_60_ and C_70_ fullerenes are the most strained, which does not prevent them to be the most stable. Obviously, their molecules have a fairly uniform strain distribution over the molecule due to the uniform distribution of pentagons. In the molecules of other fullerenes, despite their larger size, the concentration of local strains in a certain local region of a sphere is possible, causing an instability of the whole molecule. The second peculiarity of this graph is the above-mentioned “beam of stability”, inside of which all known stable IPR fullerenes are enclosed.

If the most energy favorable isomer of a given fullerene C_n_ is located on the upper line of the beam, it can be expected that experimentally it will be possible to obtain other stable isomers of this fullerene with energies determined by the distance from the upper to lower points of the beam for this fullerene. Indeed, for fullerene C_84_, this value reaches 25 kcal/mol (at that eleven isomers with a difference of up to 25–30 kcal/mol were obtained and characterized). It can be predicted that within 25 kcal/mol, several more isomers of this fullerene are likely to be obtained, including isomers with a closed shell: 6 (C_2v_), 7 (C_2v_), 12 (C_1_), 17 (C_2v_) and 21 (D_2_). At the same time, for example, for fullerene C_80_ only two isomers with an energy difference between them of just 5 kcal/mol were obtained, but their calculated energies lie at the lower border of the beam. This situation is well explained by the presented graph (Figure 11)—for those fullerenes, the most stable isomer of which lies on the lower edge of the beam (fullerene C_80_), it is not reasonable to expect experimental production of other isomers, because they will be below this area. Conversely, for the fullerenes with the most energy favorable isomer in the upper region of the beam (fullerene C_84_), the possibility of obtaining the remaining isomers becomes clear. Moreover, it is possible to expect experimental production of other isomers that meet these conditions according to the calculated energy but have not yet been obtained. It is clear that, despite a certain prognostic value, the proposed analysis, based on the integral characteristic of the molecule (expressed in values of total energies attributed to the number of carbon atoms), does not allow to judge the reasons of stability of some isomers and instability of others. Energy characteristics, geometric and electronic structures of fullerene molecules can be calculated regardless of whether they were obtained experimentally or not. However, after the analysis of the total energies (or enthalpies of formation), it is impossible to predict definitively whether a particular isomer of a given fullerene can be isolated.

To date, a large number of review papers on fullerenes have already been published, in which the main results in the field of structural studies of fullerenes, their functionalization, application prospects, etc., have been summarized. However, as mentioned above, unfortunately, there are no satisfactory criteria for assessing the stability of fullerenes and the possibility of their obtaining. The lack of a sound theoretical basis for a priori assessment of their stability often leads both theorists and experimenters to erroneous results. As a couple of examples, one can cite a theoretical study of endohedral fullerene C_84_ with methane molecule inside CH_4_@C_84_, isomer 20 (T_d_) [119], or an experimental attempt to synthesize the same isomer of fullerene C_84_ [118]. It was convincingly shown that the synthesis of this isomer cannot be successful due to its instability resulted from significant local strains in this molecule [78,79,127]. Therefore, it can be assumed that the generalized analysis of the electronic structure of already obtained (isolated and characterized) fullerenes from this point of view will reveal general regularities in their structure and, in particular, identify substructures that do not lead to destabilization of the entire molecule. Perhaps this will make it possible to identify isomers that can be obtained in the future. In addition, such analysis will provide an opportunity to reveal the regularities of the structure of unstable fullerenes.

It should be clarified here what is meant by the terms “obtained”, “extracted”, “isolated” and “characterized” (identified). If a fullerene molecule is observed in the mass spectrum of fullerene soot, it means that fullerene is obtained; then, it is transferred to a solution from soot with different solvents—extracted; then, individual isomers are isolated from the mixture of isomers. This isomer of a given fullerene can be characterized or identified based on experimental data obtained by the methods of single-crystal X-ray structural analysis, ^13^C nuclear magnetic resonance, etc., these may be small amounts sufficient only for identification; and finally, it is possible to isolate the identified fullerene isomer in an amount sufficient for its further use (e.g., chemical transformations, functionalization, etc.).

**Figure 12:**
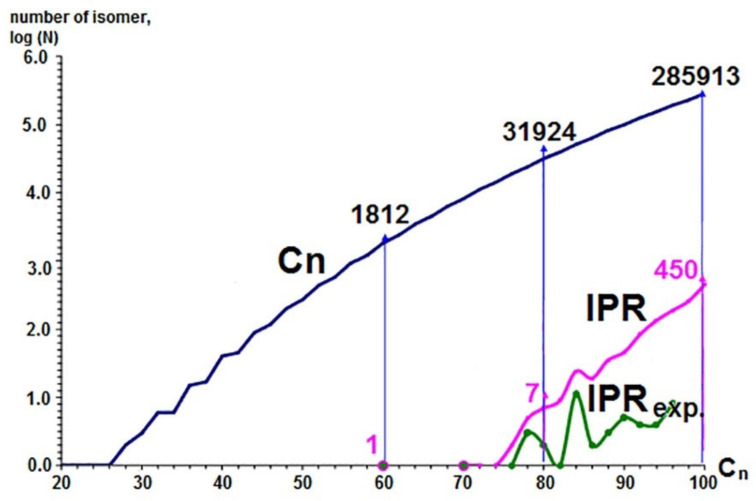
The total number of isomers (C_n_, blue curve), the number of IPR isomers (IPR, pink curve) and the number of experimentally produced, isolated and characterized IPR isomers (IPR_exp_, green curve).

The expansion of the range of fullerenes already obtained during nearly a third of a century starting with the discovery of fullerenes in 1985 is based on painstaking and extremely costly in terms of expended efforts (human, hardware) work. Nevertheless, in the interval from C_60_ to C_94,_ the number of obtained isomers does not reach 10% of the total number of fullerene IPR isomers (in the interval from C_60_ to C_84_ this value is slightly less than 30%) [35] (Figure 12).

The abovementioned problems can be solved by applying the proposed substructural approach to the modeling of fullerene structure, which allows obtaining their complete structural formula with the distribution of all types of bonds, based on which it becomes possible to judge the reasons of stability/instability of a particular fullerene molecule.

## 6. Conclusions

Thus, summarizing the above material, it is shown that the vast majority of studies are performed for fullerenes C_60_ and C_70_; however, for higher fullerenes the number of available data is much smaller. Except for the most stable fullerenes C_60_ and C_70_, it is indicative that there is no system of reference of the bonds in the molecules of higher fullerenes, traditionally accepted in organic chemistry, i.e., the localization of single, double and delocalized in hexagon π-bonds along with the fullerene shell, that significantly complicates the study of the reactivity of fullerenes and their stability.

Over the past 20 years, a structural concept of stability of the higher fullerenes has been developed. In the review, a formalization of the substructural approach for assessing the stability of higher fullerenes is proposed, which is based on a detailed analysis of the main structural features of fullerene molecules. It is based on the proposed semi-empirical approach of theoretical examination of the geometrical and electronic structure of the higher fullerenes, based on the a priori (before quantum-chemical calculations) determination of structural formula of fullerene molecule with the indication of the type of carbon-carbon bond, namely, single, double and the delocalized one. This approach was applied to a series of IPR isomers of some higher fullerenes, namely C_72_, C_74_, C_76_, C_78_, C_80_, C_82_, C_84_, C_86_. This substructural approach was fully confirmed by the subsequent quantum-chemical calculations and the entire set of experimental and theoretical studies published by research groups from different countries. This made it possible not only to predict whether which isomer of higher fullerenes would be stable but also to provide a rational explanation.

It should be noted that an important component of the substructural approach is the continuous comparison of obtained results with experimental work carried out by the world scientific community. There was a full agreement with the published experimental data, primarily the results of single-crystal X-ray structure analysis, as well as with some nuclear magnetic resonance (NMR) data. It is essential that researchers were able to identify fullerenes that cannot be obtained in the form of empty molecules and the reasons for their instability were indicated. This mainly concerns a vast group of fullerenes having an open electron shell, called radical-fullerenes. Moreover, the predictive power of the substructural approach has been tested repeatedly. For example, some isomers of fullerene C_84_, presumably designated as stable [35] were obtained two years later after theoretical work [131,132].

The developed concept of stability of higher fullerenes revealed the regularities in the structures of stable and unstable fullerenes. All stable fullerenes have structures with a closed electron shell and are characterized by the absence of excess local strains; their molecules include corannulene and indacene substructures typical for the most stable fullerenes C_60_ and C_70_; the molecules may also contain perylene and coronene substructures; the presence of three or more coronene substructures significantly destabilizes molecules of fullerenes that are at the beginning of the higher fullerene series; as the number of carbon atoms in a fullerene molecule increases, the influence of such substructures is compensated by the increase in the size of a sphere itself.

In turn, the unstable fullerenes can be divided into two groups characterized by the type of instability of the corresponding fullerene, the first and most characteristic representatives of which are fullerenes C_72_ and C_74_. The first group of fullerenes, the instability of which is characterized by an open shell and the presence of different types and numbers of radical substructures, is quite extensive. The research of recent years has shown that a significant number of such fullerenes, including non-IPR ones, are obtained as endohedral and exohedral derivatives. The instability of the latter and noticeably smaller group of fullerenes is related to the excess local strain of a molecule due to its topology. Although it is characterized by a closed shell, the presence of substructures consisting of condensed hexagons with significant local strains makes it impossible to obtain them.

## Figures and Tables

**Table 1:** HOMO-LUMO [16], criteria T [16,17] and BRE_min_ (|β|) [17,18] for some fullerenes.

Fullerene	HOMO−LUMO	T	BRE_min_
C_60_ (I_h_)	0.7566	45.40	0.082
C_70_ (D_5h_)	0.5293	37.05	0.052
C_72_ (D_6d_)	0.7023	50.57	0.0845
C_74_ (D_3h_)	0.1031	7.63	−0.0053
C_82_ isomer 1 (C_2_)	0.1495	12.26	−0.060
isomer 2 (C_s_)	0.3313	27.17	0.009
isomer 3 (C_2_)	0.256	21.06	−0.040
isomer 4 (C_s_)	0.2450	20.09	−0.031
isomer 5 (C_2_)	0.1300	10.66	−0.117
isomer 6 (C_s_)	0.0683	5.60	−0.162
isomer 7 (C_3v_)	0.0000	0.00	−0.241
isomer 8 (C_3v_)	0.0467	3.83	−0.188
isomer 9 (C_2v_)	0.0160	1.31	−0.227
C_84_ isomer 1 (D_2_)	0.6143	51.60	0.0819
isomer 20 (T_d_)	0.6962	58.48	0.0771
isomer 24 (D_6h_)	0.5293	44.46	0.0351

**Table 2:** Optimized geometric characteristics of structures with six-, five-, four- and three-membered cycles (B3LYP/631G*) [48].

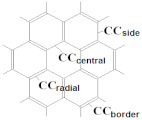	a ^1^	b	c	d
	(D_6h_)	(C_5v_)	(C_4v_)	(C_3v_)
Bonds lengths, Å				
CC_central_^2^	1.43	1.42	1.45	1.45
CC_radial_	1.42	1.38	1.37	1.37
CC_side_	1.42	1.45	1.46	1.48
CC_border_	1.37	1.39	1.38	1.38
CH	1.09	1.09	1.09	1.09
Sum of valence angles **Σ**, grad	360.00	354.07	332.17	298.88
Dihedral angles, grad.				
**I** ^3^	180.00	159.09	134.03	105.64
**II**	180.00	153.52	127.08	109.00

^1^ structures designation according to Figure 6; ^2^ bonds designations on six-membered cycle in Table heading; ^3^ **I**—dihedral angles between the planes of two hexagons, **II**—dihedral angles between the planes of the hexagon and the corresponding central cycle.

**Table 3:** Bond lengths (Å), electron affinity (eV) and HOMO-LUMO gap (eV) for phenalenyl-like structures (B3LYP/6-31G*) [65].

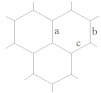	Neutral	Anion	Dianion
*C_13_H_9_—phenalenyl*			
a ^1^	1.43	1.45	1.47
b	1.39	1.39	1.42
c	1.42	1.43	1.42
Electron affinity	0.84	5.97	–
HOMO–LUMO gap	1.94	3.58	1.41
*C_13_H_6_(CH_3_)_3_—2,5,8–tri–methyl–phenalenyl*			
a	1.44	1.45	1.44–1.45
b	1.41	1.41	1.40–1.44
c	1.43–1.44	1.44	1.43–1.48
Electron affinity	0.59	5.18	–
HOMO–LUMO gap	1.80	3.29	1.35
*C_13_H_6_(C_4_H_9_)_3_—2,5,8–tri–tert–butyl–phenalenyl*			
a	1.43	1.44	1.44–1.48
b	1.40–1.41	1.40	1.40–1.45
c	42	1.42	1.41–1.44
Electron affinity	0.91	5.29	–
HOMO–LUMO gap	1.92	3.56	1.06
*C_13_H_6_(C_4_H_9_)_3_—2,5,8–tri–tert–butyl–phenalenyl (experimental values)* [56]			
a	1.41–1.42		
b	1.37–1.39		
c	1.41–1.42		

^1^ bonds designations in Table heading.

## Data Availability

Not applicable.
